# Caspase 8 and maspin are downregulated in breast cancer cells due to CpG site promoter methylation

**DOI:** 10.1186/1471-2407-10-32

**Published:** 2010-02-04

**Authors:** Yanyuan Wu, Monica Alvarez, Dennis J Slamon, Phillip Koeffler, Jaydutt V Vadgama

**Affiliations:** 1 Division of Cancer Research and Training, Department of Internal Medicine, Charles R. Drew University of Medicine and Science, (1731 East 120th Street) Los Angeles, CA (90059), USA; 2 Division of Hematology/Oncology, Department of Internal Medicine, David Geffen UCLA School of Medicine, CA (5535 Macdonald Research Laboratories Building, 675 Charles E. Young Drive South), Los Angeles, CA (90095), USA; 3 Division of Hematology/Oncology, Department of Internal Medicine, Cedars-Sinai Medical Center, (8700 Beverly Blvd., Suite 6215), Los Angeles, CA (90048), USA

## Abstract

**Background:**

Epigenetic changes associated with promoter DNA methylation results in silencing of several tumor suppressor genes that lead to increased risk for tumor formation and for progression of the cancer.

**Methods:**

Methylation specific PCR (MSP) and bisulfite sequencing were used for determination of proapoptotic gene Caspase 8 (CASP8) and the tumor suppressor gene maspin promoter methylation in four breast cancer and two non-tumorigenic breast cell lines. Involvement of histone H3 methylation in those cell lines were examined by CHIP assay.

**Results:**

The CpG sites in the promoter region of CASP8 and maspin were methylated in all four breast cancer cell lines but not in two non-tumorigenic breast cell lines. Demethylation agent 5-aza-2'-deoxycytidine (5-aza-dc) selectively inhibits DNA methyltransferases, DNMT3a and DNMT3b, and restored CASP8 and maspin gene expression in breast cancer cells. 5-aza-dc also reduced histone H3k9me2 occupancy on CASP8 promoter in SKBR3cells, but not in MCF-7 cells. Combination of histone deacetylase inhibitor Trichostatin A (TSA) and 5-aza-dc significant decrease in nuclear expression of Di-methyl histone H3-Lys27 and slight increase in acetyl histone H3-Lys9 in MCF-7 cells. CASP8 mRNA and protein level in MCF-7 cells were increased by the 5-aza-dc in combination with TSA. Data from our study also demonstrated that treatment with 5-FU caused a significant increase in unmethylated CASP8 and in CASP8 mRNA in all 3 cancer lines.

**Conclusions:**

CASP8 and maspin expression were reduced in breast cancer cells due to promoter methylation. Selective application of demethylating agents could offer novel therapeutic opportunities in breast cancer.

## Background

Aberrant DNA methylation has been recognized as one of the most common molecular alterations in human neoplasia. Hypermethylation of gene-promoter regions is being revealed as one of the most frequent event that causes loss of gene function. DNA methylation usually occurs at a cytosine associated with CpG sites [1]. DNA (cytosine-5)-methyltransferase (DNA-MTase) catalyzes this reaction by adding a methyl group from S-adenosyl-L-methionine to the fifth carbon position of the cytosine [1]. Methylation of CpG sites in the promoter region of the genes is known to transcriptionally repress these genes [2]. CpG sites of a large number of genes that are unmethylated in normal tissue are methylated in human cancers, such as breast, ovarian, colon, and prostate cancers [3,4]. Methylation at the promoter region of specific genes depends on tumor type. For example, the mismatch repair gene hMLH1 is silenced by hypermethylation more frequently in colorectal, endometrial, and gastric tumors; while the BRCA1 is methylated in breast and ovarian tumors [5-8]. Recent studies have suggested that CpG methylation of certain genes may be associated with HER2 receptor overexpression and/or hormone status in breast cancer [8,9]. It is unclear as to which breast cancer specific genes are transcriptionally silenced and if their silencing is associated with failure in treatment and decrease in disease-free survival (DFS).

CASP8 is an important initiator of apoptosis [10]. Absent or downregulation of CASP8 could cause resistance to apoptosis and is correlated with unfavorable disease outcome, such as in childhood medulloblastoma and neuroblastoma [11,12]. The absence or downregulation of CASP8 may be due to epigenetic changes. Studies have also indicated that methylation and demethylation of maspin promoter may regulate maspin gene expression and that reduced maspin expression is associated with cancer progression [13].

In the current study we used methylation specific PCR (MSP), and bisulfite sequencing to determine the methylation status of these two genes. We examined the mechanisms associated with transcriptional silencing of CASP8 and maspin by promoter methylation using real-time PCR and by restoring the methylated genes back to their unmethylated status using the demethylating agent, 5-aza-2'-deoxycytidine; TSA (Trichostatin A), inhibitor of histone deacetylase; and chemotherapeutic agent 5-Fu (5-Fluorouracil).

## Methods

### Cells and culture

The breast cancer cells with varying tissue subtypes selected for our methylation studies were: MCF-7 (ER positive and HER2/neu negative); MDA-MB231 (ER negative and HER2/neu negative); SKBR3 (ER negative and HER2/neu positive); HCC1937 (ER negative, HER2/neu negative and BRCA1 mutated); non-tumorigenic breast epithelial cells (MCF12A), and non-tumorigenic breast fibroblast cells (MCF10). These cell lines were purchased from American Type Culture Collection (Rockville, MD), and unless otherwise stated, the cells were grown and maintained in DMEM/F12 (Fisher Scientific, CA) containing 10% FCS (Invetrogen), 2 mM glutamine, 50 units/ml penicillin and 50 μg/ml streptomycin (Fisher Scientific, CA).

### 5-aza-2'-deoxycytidine (5-aza-dc) and Trichostatin A (TSA) treatment

The cells were growing in culture medium until 80% confluence; 5 μM 5-aza-dc was added and incubated for 3 to 6 days. The medium containing 5-aza-dc was refreshed every 2 days. For combination treatment cells were first treated with 0.3 μM TSA in combination with 5 μM 5-aza-dc for 2 days and then TSA was removed from culture medium. Treatment with 5 μM 5-aza-dc was continued for 1 more day.

### Bisulfite modification and Methylation Specific PCR (MSP)

Bisulfite conversion of genomic DNA was carried out using Zymo EZ DNA Methylation-Gold™ kit (D5005, Zymo Research Corp, Orange, CA) according to the manufacture's instructions. This process converts unmethylated cytosine residues to uracil, while methylated cytosine residues remain unchanged. Bisulfite modified DNA was used as a template, and then MSP was performed to determine the methylation status of CASP8 and maspin. The primer sequences for MSP are as follows:

(a) CASP8, 5'-TGTTGTTTGGGTAACGTATCGA-3' (methylated forward),

5'-CCCTACTTAACTTAACCCTACTCGAC-3' (methylated reverse), and

5'-TTGTTGTTTGGGTAATGTATTGA-3' (unmethylated forward),

5'-CAACCCTACTTAACTTAACCCTACTCA-3' (unmethylated reverse);

(b) maspin, 5'-ATTTTATCGAATATTTTATTTTTCGG-3' (methylated forward),

5'-TAACTCACCTAAACAACACCGCC-3' (methylated reverse), and

5'-TTTTATTTTATTGAATATTTTATTTTTTGG-3' (unmethylated forward),

5'-TAACTCACCTAAACAACACCACC-3' (unmethylated reverse).

The primers for MSP were designed using MethPrimer [14] according to the sequences provided by Panomics (Fremont, CA). The cover regions were shown in Additional file 1, Figure S1. PCR conditions were as follows: an initial denaturation at 95°C for 5 min was followed by 40 cycles at 94°C for 30 s, 50°C for 30 s, and 72°C for 45 s and a final extension at 72°C for 7 min.

### Bisulfite sequencing for CASP8 promoter

Bisulfite modified DNA (described above) was amplified by two primer sets. The primer sequences were as follows: P1-CASP8 (nt -744 to nt -466), 5'-GGTGGTGGGTGTTTGTAGTTTTAGT-3' (forward) and 5'-CCATCCTTAACCATATTCTCCAATTTA-3' (reverse); P2-CASP8 (nt -486 to nt +51), 5'-TAGATTTTTTGTAAGAAAGAATGGTAT-3' (forward) and 5'-ACAAAAAAACAAAATCTAATCTCC-3' (reverse). PCR conditions were as follows: an initial denaturation at 95°C for 5 min was followed by 40 cycles of 94°C for 30 s, 52°C for 30 s, and 72°C for 45 s and a final extension at 72°C for 7 min. The PCR products were purified with QIAquick PCR purification kit (#28104, QIAGEN), and then sent to Retrogen Inc (San Diego, CA) for sequencing. The primers for bisulfite sequencing were designed using MethPrimer [14]. The sequence was used to design the bisulfate sequence primers were as same as the sequence used to design primers for MSP. The specific cover regions for each primer site have been demonstrated in Additional file 1.

### Quantitative real-time PCR (Q-PCR)

Total RNA from cultured cells was isolated by using RNeasy micro kit (#74004, QIAGEN) according to the manufacture's instructions, and cDNA was synthesized by reverse transcription (RT) with ThermoScript™ RT-PCR system (Invitrogen) according to the manufacture's instructions. Q-PCR analysis was performed with iCycle iQ real-time PCR detection system (Bio-Rad Lab, Hercules, CA) using SYBR Green Master Mix (#204143, QIAGEN). The primers used were as follows: (a) CASP8, 5'-CCAGAGACTCCAGGAAAAGAGA-3' (forward) and 5'-GATAGAGCATGACCCTGTAGGC-3' (reverse). (b) maspin, 5'-AGATGGCCACTTTGAGAACATT-3' (forward) and 5'-GGTTTGGTGTCTGTCTTGTTGA-3' (reverse). (c) housekeeping control gene, 18 s, 5'-GATCCATTGGAGGGCAAGTC-3' (forward) and 5'-TCCCAAGATCCAACTACGAG-3' (reverse). Reactions were characterized during cycling when amplification of the PCR product was first detected (*C_t_*). The target message and the housekeeping gene, 18 s, in breast cancer and non-tumorigenic cell lines, were quantified by measuring *C_t_*. The relative level of target messages in cells was normalized on the basis of its 18 s content by taking the difference of threshold cycles between target gene and 18 s. Using non-tumorigenic breast cancer cells or untreated breast cancer cells as reference, the level of CASP8 and maspin in each sample was normalized with the corresponding reference by taking the difference between threshold cycles. Final results were expressed as N-fold difference in target gene expression relative to the reference.

### Immunobloting and Immunofluorescence (IF)

For immunoblot analysis cells were lysed in 1 × lysis buffer (20 mM Tris pH 7.5; 150 mM NaCl; 1 mM EDTA; 1 mM EGTA; 1% Triton X-100; 2.5 mM sodium pyrophosphate; 1 mM β-glycerolphosphate; 1 mM Na_3_VO_4_; 1 μg/ml leupeptin; 0.1 mM PMSF) and protein concentration was measured using BCA dye (Pierce). Total protein (50 μg) from cell lysates was resolved on SDS-PAGE followed by transfer to nitrocellulose membrane. The membranes were incubated with antibodies specific against Dnmt1 (IMG-261A, IMGENEX), Dnmt2 (IMG-281, IMGENEX), Dnmt3a (IMG-268A, IMGENEX), Dnmt3b (IMG-184A, IMGENEX), and caspase-8 (#9746, Cell Signaling) according to manufacturer's instruction. Detection of antigen-bound antibody was performed with the enhance chemiluminescence reagent. For immunofluorescence analysis cells (2 × 10^4^/each) were mounted by cytospin on polylysine coated glass slides and fixed with 4% paraformaldehyde for 15 min followed by 100% ice-cold acetone for 10 min at 4°C. To detect caspase-8 protein expression immunofluorescence analysis was performed with cleaved caspase-8 antibody (#9496, Cell Signaling) followed by incubation with anti-goat IgG-FITC (Santa Cruz) for 30 min in dark and mounted with DAPI mounting medium (Vector Labs). The cells with positive staining were counted in five different areas and adjusted with total number of cells. To analyze histone H3 methylation and acetylation status in MCF-7 cells double immunofluorescence analysis was performed with antibodies against Di-Methyl Histone H3 (Lys27) (#9755, Cell Signaling) or Acetyl Histone H3 (Lys9) (#9671, Cell Signaling) followed by β-actin antibody (A1975, Sigma-Aldrich). FITC-conjugated secondary antibodies were used to label Di-Methyl Histone H3 (Lys27) and Acetyl Histone H3 (Lys9). The β-actin was labeled with Tex-red-conjugated secondary antibody. After mounting, the cells were viewed under a fluorescence microscope.

### CHIP assay

Cells were fixed with formaldehyde (1% final concentration) to cross-link protein to DNA at room temperature for 10 min after treated with or without 5-aza-dc (5 μM, 3 days), and then incubated with glycine (0.125 M final concentration) for 5 min to stop the cross-linking. Soluble chromatin was prepared as described previously [15]. The chromatin was then diluted 1:10 with dilution buffer (0.01% SDS, 1.1% Triton X-100, 1.2 mM EDTA, 16.7 mM Tris-HCl, 167 mM NaCl and a protease inhibitor cocktail set (CalBiochem)), and subjected to immunoprecipitation with anti-dimethylated histone H3 lysine 9 (H3K9me2) antibody (#9753, Cell signaling). Pre-immune serum (Santa Cruz) was also used as a control. The chromatin and antibodies were incubated overnight at 4°C. Chromatin/antibody complexes were recovered by adding 30 μl of protein A/G Plus-agarose beads (Santa Cruz) and incubating at 4°C for 2 h. The beads were sequentially washed for 10 min each in 1 ml of low salt, high salt and LiCl immune complex wash buffer. Immunocomplexes were eluted off the beads by incubation with 300 μl of 1% SDS and 50 mM NaHCO_3_. The eluent was incubated at 65°C for 5 h or overnight to reverse the formaldehyde-induced protein-DNA cross-links. The DNA was extracted with phenol and chloroform. Extracted DNA was resuspended in 100 μl of TE buffer and real time PCR was performed by using Bio-Rad thermal cycler with CASP8 primers: forward 5'GGT GCC TGT AGT CCC AGC TAC TC3' and reverse 5'CCT AGA CCC TCC CCT GTT CTG TC3'. For input DNA, the chromatin preparation without incubated with antibodies was subjected to real time PCR.

## Results

### Promoter methylation on CASP8, and maspin resulted in decrease mRNA and protein expression

**Figure 1:**
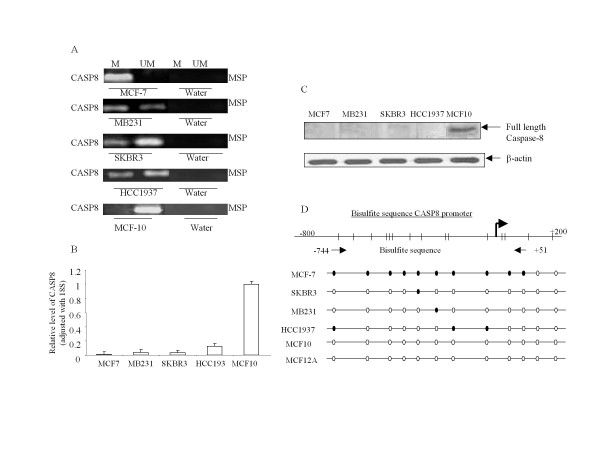
**CASP8 promoter methylation resulted in decreased mRNA and protein expression**. (**A**) DNA from the indicated cell lines were modified by bisulfite treatment, and MSP were performed with methylated and unmethylated CASP8 PCR primers as described in Methods. The bands detected by methylated primer represented methylated CASP8 (M), and the bands detected by unmethylated primer represented unmethylated CASP8 (UM). (**B**) RNA were isolated from cells and performed for RT and real-time PCR with CASP8. The relative level of CASP8 gene was normalized to 18 s mRNA as described in Methods. Each bar represented the mean of three independent amplifications with standard deviation (SD). (**C**) Total protein was isolated from the indicated cells, and Western blot analysis was performed with antibodies specific for full length caspase-8. β-actin was used as loading control. (**D**). Bisulfite modified DNA from the indicated cells were amplified with CASP8 primer designed according to the bisulfite modified sequence of CpG sites in CASP8 promoter as described in Methods. The amplified PCR products were purified and sequenced to confirm the location of methylated CpG sites. The dark dots represent location of methylated CpG sites in the promoter, and open dots indicate unmethylated CpG sites.

First, we determined the promoter methylation status in four breast cancer and one non-tumorigenic breast cell lines by MSP. We then examined if the promoter methylation would silence or decrease the gene or protein expression for CASP8 and maspin in different breast cancer and non-tumorigenic breast cells, and asked whether the 5-aza-cd treatment would reverse the methylation and change the mRNA expression of these genes. Results from MSP showed that CASP8 promoter was methylated in breast cancer cells, MCF-7, MB231, SKBR3, and HCC1937; but not in non-tumorigenic breast cells, MCF-10 (Figure 1A). The CASP8 promoter methylation resulted in the gene silence and lack of CASP8 in those breast cancer cells. CASP8 mRNA level were decreased significantly in all four breast cancer cells compared to non-tumorigenic breast cells, MCF-10 (Figure 1B). All four breast cancer cell lines, MCF-7, MB231, SKBR3 and HCC1937, lacked protein expression of caspase-8 (Figure 1C). Bisulfite sequence analysis demonstrated the position of the methylated CpG sites of CASP8 in different breast cancer cells (Figure 1D).

**Figure 2:**
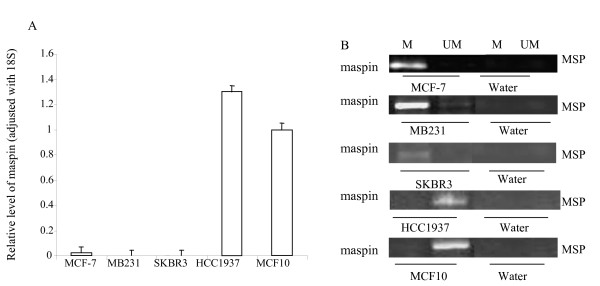
**Maspin promoter methylation resulted in decreased mRNA and protein expression**. (**A**) RNA were isolated from cells and performed for RT and real-time PCR with maspin primers. The relative level of the maspin gene was normalized to 18 s as described in Methods. Each bar represented the mean of three independent amplifications with standard deviation (SD). (**B**) DNA from the indicated cell lines were modified by bisulfite treatment, and MSP were performed with methylated and unmethylated maspin PCR primers as described in Methods. The bands detected by methylated primer represented methylated maspin (M), and the bands detected by unmethylated primer represented unmethylated maspin (UM).

The mRNA level of maspin was decreased in MCF-7 cells; it was undetectable in MB231 and SKBR3 and remained high level in HCC1937 compared to MCF10 cells (Figure 2A). Consistent with the mRNA expression, results from MSP demonstrated that the maspin promoter in MCF-7, MB231 and SKBR3 were methylated (Figure 2B).

### Effect of 5-aza dc on DNA methylation

**Figure 3:**
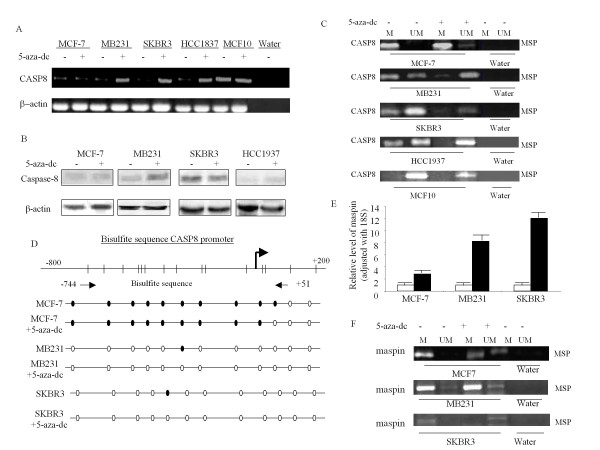
**Effect of 5-aza-dc treatment on mRNA, protein expression and promoter methylation of CASP8 and maspin**. Cells were treated with 5 μM 5-aza-dc for 3 days. (**A**) RNA were isolated from the treated and untreated cells and performed for RT-PCR with CASP8 primers. β-actin was used as loading control. (**B**) Total protein was isolated after cells were treated or untreated with 5-aza-dc. Western blot analysis was performed with antibodies specific for full length caspase-8, and β-actin was used as loading control. (**C**) DNA were isolated and modified by bisulfite treatment. MSP was performed with methylated and unmethylated CASP8 PCR primers as described in Methods. The bands detected by methylated primer represented methylated maspin (M), and the bands detected by unmethylated primer represented unmethylated maspin (UM). (**D**) The DNA from 5-aza-dc treated and untreated cells were modified by bisulfite treatment and amplified with CASP8 primer designed according to the bisulfite modified sequence of CpG sites in CASP8 promoter as described in Methods. The amplified PCR products were purified and sequenced for confirmation the CpG sites methylation. The dark dots represented methylated CpG sites in the promoter, and open dots indicated unmethylated CpG sites. (**E**) RNA from 5-aza-dc treated and untreated cells were used to perform RT and real-time PCR with maspin primers. The relative level of maspin gene was normalized to 18 s as described in Methods. Each bar represented the mean of three independent amplifications with standard deviation (SD). (**F**) DNA from cells treated and untreated with 5-aza-dc were modified by bisulfite treatment, and MSP was performed with methylated and unmethylated maspin PCR primers as described in Methods. The bands detected by methylated primer represented methylated maspin (M), and the bands detected by unmethylated primer represented unmethylated maspin (UM).

Next, we examined if the mRNA expression of CASP8, and maspin could be restored by demethylating the target genes. We treated MCF-7, SKBR3, and MB231 with 5 μM 5-aza-dc for 3 days and performed RT-Q-PCR. Figure 3A confirms that gene expression of CASP8 in MB231, SKBR3, and HCC1937 cells can be restored with 5-aza-dc treatment. The protein expression of CASP8 in MB231, SKBR3, and HCC1937 was also restored with 5-aza-dc treatment (Figure 3B). The gene and protein expression of CASP8, however, were not restored with 5-aza-dc treatment for 3 days in MCF-7 cells (Figure 3A and 3B).

To further confirm our hypothesis that loss of CASP8 in breast cancer cells may be due to promoter methylation, we performed MSP, which detected methylation of CpG sites of CASP8 in 5-aza-dc untreated breast cancer cells, MCF-7, MB231, SKBR3, and HCC1937 but not in MCF10 (Figure 3C). MSP also detected unmethylated CASP8 in MB231, SKBR3, and HCC1937 untreated cells (Figure 3C). However, as shown in Figure 3C, 5-aza-dc treatment completely demethylated the CASP8 promoter in MB231, SKBR3, and HCC1937 cells. In contrast, after treatment with 5-aza-dc, MSP detected a weak band of unmethylated CASP8 in MCF-7 cells. A good amount of methylated CASP8 still remained methylated after 5-aza-dc treatment in MCF-7 cells (top panel of Figure 3C). To determine the optimal time for treatment with 5-aza-dc to induce demethylation of CASP8 in MCF-7, cells were treated with 5-aza-dc from 3 to 6 days. MSP and RT-Q-PCR were used to assess the extent of demethylation. There were no significant changes in CASP8 promoter methylation as well as in mRNA expression after 3 days treatment (data not shown). However, after four days of treatment with 5-aza-dc, we did see some restoration of unmethylated CASP8. The CASP8 mRNA expression also increased further after four days of treatment.

Taken together, the results from MSP confirmed that the loss of CASP8 gene and protein expression in these breast cancer cells resulted from CASP8 promoter methylation. Results also confirmed that CASP8 gene and protein can be restored by demethylation in selected breast cancer cells. Bisulfite sequencing analysis further demonstrated the position of demethylation of CASP8 related to restoring CASP8 gene and protein levels in 5-aza-dc treated cells (Figure 3D).

5-aza-dc treatment also reactivated gene expression of maspin in MCF-7, MB231, and SKBR3 (Figure 3E). MSP confirmed that the reactivated maspin gene expression with 5-aza-dc treatment was consistent with demethylation on maspin promoter (Figure 3F). The CpG sites on maspin promoter in HCC1937 cells were not methylated; hence, we did not perform RT-Q-PCR and MSP for maspin on HCC1937 cell treated with 5-aza-dc. The increased gene expression following demethylation suggested that methylation-dependent transcriptional silencing may cause decreased expression or even total loss of CASP8 and maspin in breast cancer cells.

### Effect of 5-aza-dc on DNA Methyltransferases

**Figure 4:**
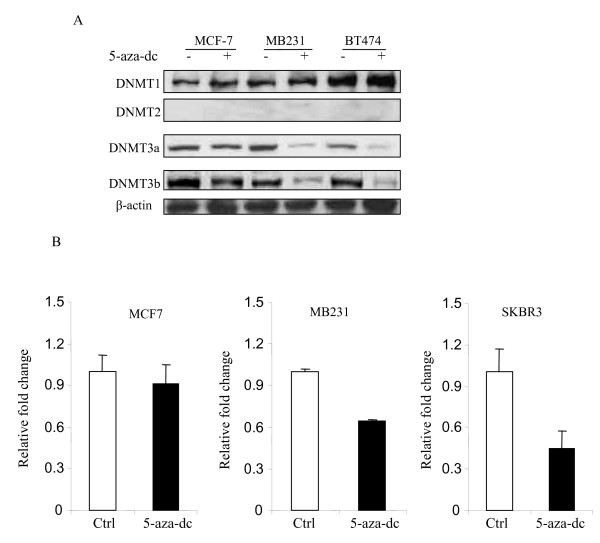
**Effect of 5-aza-dc on DNMTs, and H3K9me2**. (**A**) Total protein from the 5-aza-dc treated and untreated cells was used to perform Western blot analysis with antibodies specific against DNMT1, DNMT2, DNMT3a and DNMT3b as described in Methods. Antibody against β-actin was also used as loading control. (**B**) Chromatins were extracted from cells with or without 5-aza-dc treatment as described in Methods. Chromatin immunoprecipitation was performed with antibody specific against histone H3K9me2, and recovered DNA fragments were used for Q-PCR with promoter specific primers of CASP8. Fold change of H3K9me2 occupancy was calculated based on the control (without 5-aza-dc treatment). Each bar represented the mean of three independent amplifications with standard deviation (SD).

Since, 5-aza-dc (inhibitor of DNA methyltransferase) treatment did not demethylate CASP8 promoter region, we examined if 5-aza-dc was able to inhibit DNMTs (DNA methyltransferases). Figure 4A shows that 5-aza-dc treatment did not inhibit DNMT1, DNMT3a and DNMT3b in MCF-7 cells. However, it did inhibit DNMT3a and DNMT3b in MDAMB231 and BT474 (ER positive and HER2 positive) breast cancer cells. We did not detect DNMT2 in any of the cell lines tested. In addition, DNMT1 was not inhibited by 5-aza-dc in all three breast cancer cells tested.

### CHIP Assay to determine the specificity of CASP8 demethylation in response to 5-aza-dc

DNA methyltransferase inhibitors commonly used in clinical trials promote tumor cell death, but their detailed cytotoxic action is not yet fully understood. A deeper knowledge about their apoptosis-inducing mechanisms and their interaction with DNA methyltransferases (DNMTs) DNMT1, DNMT3a, and DNMT3b might allow the design of more effective drugs with lower cytotoxicity. 5-aza-deoxycytidine (5-aza-dc), a potent inhibitor of DNMT1, is known to induce demethylation and reactivation of silenced genes. Our data suggest that 5-aza-dc treatment increases CASP8 transcription in MB231, SKBR3 and BT474, but not in MCF-7 cells. Recent studies indicate that histone H3 lysine methylation represses gene transcription [16,17]. Thus, we further investigated whether 5-aza-dc reduces histone H3 methylation to activate CASP8 transcription in breast cancer cells. Our results (Figure 4B) show that 5-aza -dc inhibits H3K9me2 occupancy on CAPS8 promoter in SKBR3 and MB231, but not in MCF-7 cells lines. These results suggest that CASP8 gene transcription is restored in theses SKBR3 and MB231, but not in MCF-7 cells.

### Effect of inhibiting HDAC with TSA

**Figure 5:**
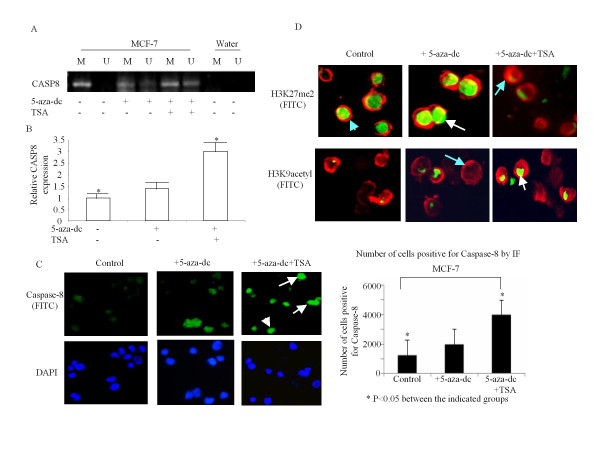
**Effect of TSA in combination with 5-aza-dc on CASP8**. (**A**) DNA was isolated from the TSA and 5-aza-dc treated and untreated cells and MSP was performed with methylated and unmethylated CASP8 PCR primers as described in Methods. The bands detected by methylated primer represented methylated CASP8 (M), and the bands detected by unmethylated primer represented unmethylated CASP8 (UM). (**B**) RNA was isolated from the TSA and 5-aza-dc treated and untreated cells. RT and Q-PCR was performed with CASP8 primers. The relative level of CASP8 mRNA was normalized to 18 s as described in Methods. Each bar represented the mean of three independent amplifications with standard deviation (SD), *p < 0.05 between indicated groups. (**C**) Immunofluorescence analysis was performed with cleaved caspase-8 antibody followed by incubation with anti-goat IgG-FITC and mounted with DAPI mounting medium (left panel). The arrows indicate the FITC labeled cleaved caspase-8 (green in the top panel of left), and the cell nucleus was labeled by DAPI (blue in bottom panel of left). The cells with positive staining for cleaved caspase-8 were counted in five different areas and adjusted with total number of cells (right panel). Each bar represents the mean of number of cells positive for cleaved caspase-8 in the five areas with standard deviation (SD), * p < 0.05 between the indicated groups. (**D**) Double immunofluorescence analysis was performed with Di-Methyl Histone H3 (Lys27) or Acetyl Histone H3 (Lys9) antibodies followed by actin antibody as described in Methods. The nucleus positive stained with Di-Methyl Histone H3 (Lys27) or Acetyl Histone H3 (Lys9) were labeled with FITC as green (indicated by white arrows), and the cytoplasm stained with actin was labeled with Tex-red as red (indicated with light blue arrows).

Histone deacetylation is an important mechanism of regulation of gene expression. Since MCF-7 cells treatment with 5-aza-dc did not increase the levels of CASP8 gene and protein expression, we also investigated whether treatment of MCF-7 cells with TSA (Trichostatin A), inhibitor of histone deacetylase (HDAC), in combination with 5-aza-dc could change the methylation status of CASP8 and alter the gene and protein expression. As shown in Figure 5A, MCF-7 treated with TSA (0.3 μM for 2 days) in combination with 5-aza-dc partially demethylated CASP8 promoter. The mRNA level of CASP8 was significantly increased (Figure 5B), and the cleaved caspase-8 protein could be seen in cells treated with TSA in combination with 5-aza expression (Figure 5C). Consistent with the mRNA and protein data, TSA in combination with 5-aza-dc treatment significantly reduced Di-Methylated Histone H3 (Lys27) and increased Acetylated Histone H3 (Lys9), as shown in Figure 5D.

### Effect of 5-Fu on CASP8

**Figure 6:**
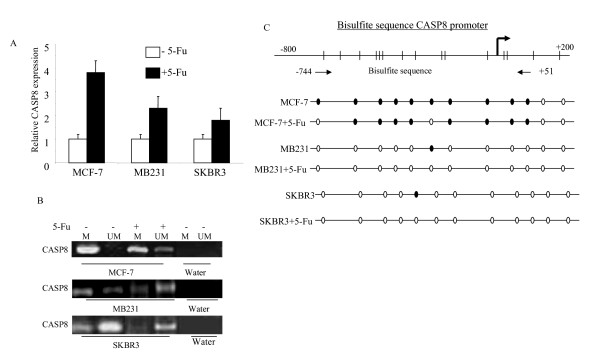
**Effect of 5-Fu on CASP8**. (**A**) MCF-7, MB231, and SKBR3 cells were treated with or without 5-Fu (10 μM) for 3 days. RNA was isolated from the treated and untreated cells and RT-real-time PCR was performed with CASP8 and 18 s primers. Each bar represented the mean relative level of CASP8 and standard deviation (SD) from three measurements and normalized to 18 s. (**B**) DNA was isolated from the 5-Fu treated and untreated cells and modified with bisulfite treatment. MSP was performed with methylated and unmethylated CASP8 primers. The bands detected with methylated primer indicated methylated CASP8 (M), and the bands from unmethylated primer indicated unmethylated CASP8 (UM). (**C**) Bisulfite modified DNA from the indicated 5-Fu treated, and untreated cells were amplified with CASP8 primer designed according to the bisulfite modified sequence of CpG sites in CASP8 promoter as described in Methods. The amplified PCR products were purified and sequenced for confirmation of the CpG sites methylation. The dark dots in the bottom panel represented methylated CpG sites in the promoter, and open dots indicated unmethylated CpG sites.

Recent studies have shown that the anticancer drug, 5-fluorouracil (5-Fu), can upregulate CASP8 protein and induce cell apoptosis in leukemia and breast cancer cells [18,19]. 5-Fu has been successfully used for treatment of breast cancer in clinical practice. We examined if 5-Fu treatment could upregulate CASP8 in breast cancer and if the upregulation involved demethylating CpG sites on CASP8 promoter. As shown earlier, CASP8 mRNA was undetectable in MCF-7 breast cancer cells due to promoter methylation. We then treated MCF-7 cells with 5-Fu for 3 days and examined CASP8 mRNA levels. Figure 6A shows almost 4 fold increase in mRNA level in MCF-7 cells treated with 5-Fu compared to non-treated cells. The mRNA level of CASP8 in MB231 and SKBR3 treated with 5-Fu also increased 2.4 fold and 1.8 fold respectively (Figure 6A). The increases in mRNA expression are correlated with partial demethylation of CpG sites on CASP8 promoter (Figure 6B). Bisulfite sequencing confirmed the position (nt -642 to nt -532, located in CASP8 core promoter region) of demethylation of CASP promoter by 5-Fu in these breast cancer cells (Figure 6C).

## Discussion

Hypermethylation of the promoter regions of various genes has been recognized as one of the most frequent mechanisms causing loss of gene function. The association between aberrant DNA methylation and carcinogenesis has been demonstrated in several studies [20-26]. However, the association between epigenetic changes with cancer etiology needs to be elucidated.

Several cancer-related genes have been reported to be silenced by aberrant methylation in breast cancer, such as 14-3-3 s, E-cadherin and tissue inhibitor of metalloproteinase 3 (TIMP3) genes. Treatment with 5-aza-dc activated the expression of 14-3-3 s [27] and E-cadherin genes [28] in breast carcinoma cells and of TIMP3 in different tumor cell lines [29,30].

Hypermethylation of CASP8 has been showed as a frequent feature of relapsed glioblastoma compared with the corresponding primary tumors [31]. The authors suggested that epigenetic deregulation of the mitochondria-independent apoptosis is a relevant characteristic in recurrent glioblastoma. The development of targeted therapies restoring functional extrinsic apoptosis, as recently shown *in vivo *with the synergistic combination of the DNA demethylating agent decitabine and TRAIL [32], may provide a useful tool to overcome the resistance of glioblastoma to contemporary treatment modalities. Methylation of CASP8 gene has also been reported in some childhood tumors and in neuroendocrine lung tumors [33]. CASP8 is an important initiator of apoptosis [10]. Structurally, the promoter region of CASP8 has binding sites for p53, nuclear factor-κB (NF-κB), AP-1, SP-1, IRF-1, and Ets-like transcription factors [34]. Therefore, CASP8 functions both as a pivotal molecule for death-receptor-induced apoptosis and as a selective signal transducer, such as for NF-κB activation [35]. Absent or downregulation of CASP8 could cause resistance to apoptosis and is correlated with unfavorable disease outcome, such as in childhood medulloblastoma and neuroblastoma [11,12]. Others have also demonstrated that absence or downregulation of CASP8 may be due to epigenetic changes, such as hypermethylation, or mutations [36,37].

In current study we have investigated the promoter methylation of CASP8 and maspin in relation to their expression levels as well as the involvement of histone methyltransferases and histone H3K9me2. Using MSP and bisulfite sequence analysis, we have established the relationship between aberrant cytosine methylation and downregulated or loss of CASP8 in breast cancer cells. We confirmed that CpG sites methylation in the promoter region of CASP8 is the mechanistic basis for transcriptional downregulation or silencing of CASP8 in breast cancer cells. The methylation status of CASP8 can be completely or partially reversed by treatment with 5-aza-dc in MB231, SKBR3, and BT474, but not in MCF-7 breast cancer cells. The cells that had fewer methylated CpG sites, such as MB231 and SKBR3 were totally demethylated by 5-aza-dc. This change in demethylation resulted in a significant increase in CASP8 mRNA and protein expression. In contract SKBR3 cells, most CpG sites of CASP8 were methylated in MCF-7 cells. Results from MSP showed that methylation was partially reversed by 5-aza-dc in MCF-7 cells and the mRNA and protein level of CASP8 had no significant increase. We also examined the effect of histone acetylation of CASP8 by treating MCF-7 cells with Trichostatin A (TSA). The TSA treatment alone did not change the methylation status and mRNA expression of CASP8 in MCF-7 cells (data not shown). However, TSA in combination with 5-aza-dc was able to partially demethylate CASP8 promoter and increased CASP8 mRNA and protein expression in MCF-7 cells. The data suggested the involvement of histone acetylation in the regulation of CASP8 gene expression in MCF-7 cells. CHIP analysis (Figure 4B) showed that 5-aza-dc treatment inhibits H3K9me2 occupancy on CAPS8 promoter in SKBR3 and MDA-MB231, but not in MCF-7 cells. These results suggest that CASP8 gene transcription is restored in theses SKBR3 and MB231, but not in MCF-7 cells.

Several studies have demonstrated a link between methylation and histone acetylation in which a family of methyl-CpG-binding proteins is involved [38]. When these proteins bind to a methylated promoter, they recruit HDAC, and the interaction of these two epigenetic events inhibits gene expression by interfering with the function of transcription factors and the compaction of the chromatin structure [39-41]. Inhibitors of these epigenetic changes can lead to the reactivation of genes that suppress tumorigenesis. In accord with this hypothesis is the report on the synergistic interaction of 5-aza-dc and the HDAC inhibitor, trichostatin A (TSA), in the reactivation of tumor suppressor genes [42]. This same drug combination was also reported to induce a synergistic reactivation of the estrogen receptor-α in breast carcinoma cells [43].

A recent study in prostate cancer cells indicated that CASP8 plays a pathway specific role in inhibiting androgen receptor signaling [44]. This evidence suggests that CASP8 may have the role beyond its role as a cell death protease and may play a role in hormonal receptor cell signaling.

The anticancer drug, fluorouracil (5-Fu), has been commonly used in clinical practice for first line treatment of breast cancer for decades. Up to now, except for interferon-γ and azacytidine, the cytotoxic drugs, 5-Fu and methotrexate, have been shown to upregulate CASP8 and induce cell apoptosis in neuroblastoma, medulloblastoma, Ewing sarcoma, glioblastoma, leukemia, and breast cancer cells [18,19]. The mechanism by which 5-Fu regulates CASP8 protein is more likely to involve p53 [19]. However, studies from Zoli, et al. [18] suggest that 5-Fu in combination with doxorubicin and paclitaxel regulates CASP8 and induces cell apoptosis by a caspase-dependent mechanism independent of hormonal, p53, bcl-2 or bax status in breast cancer cells [18]. These observations made us to suspect that 5-Fu induced cell apoptosis may involve a demethylation process. We then tested the expression CASP8 mRNA, as well as its methylation status, in MCF-7, MB231 and SKBR3 cells treated with 5-Fu for 3 days. Compared to untreated cells, the cells treated with 5-Fu showed a significant increase mRNA expression of CASP8 followed by demethylation of its promoter region.

Taken together, our results confirm that expression of CASP8 in breast cancer is epigenetically controlled and the modification may vary in different types of breast cancer cells.

Another gene promoter methylation has been studied was maspin in current study. Maspin belongs to the serpin family [45]. It acts as a tumor suppressor, increases cell adhesion, induces apoptosis, and inhibits tumor growth and metastasis [46-48]. Maspin is also involved in angiogenesis and mammary gland development [49]. The expression of maspin is epigenetically controlled by methylation and/or histone acetylation. Studies have also indicated that methylation and demethylation of maspin promoter may regulate maspin gene expression and that reduced maspin expression is associated with cancer progression [13]. In our study, we have confirmed that the expression of maspin in breast cancer cells is epigenetically controlled by methylation of the CpG sites. The demethylation agent, 5-aza-dc, treatment reversed maspin promoter methylation and increased maspin gene expression in MCF-7, MB231, and SKBR3 cells. An early study from Domann FE et al. [50] also reported that normal human mammary epithelial cells expressed maspin mRNA and displayed a completely non-methylated maspin gene promoter. In contrast, most breast cancer cell lines had no detectable maspin expression and those maspin-negative breast cancer cell lines also displayed an aberrant pattern of cytosine methylation of the maspin promoter. In this study we have also examined maspin and CASP8 mRNA levels in 30 breast cancer tissues and 10 non-breast cancer tissues. The mRNA levels of CASP8 and maspin were lower in breast cancer tissue than non-breast cancer tissue (data not shown). The decreased mRNA expressing levels of maspin and CASP8 in patients were associated with positive lymph node status, late stage disease, and HER2 overexpression (3+ and/or FISH positive). The DFS was significant decreased in patients with low CASP8 or maspin expression (data not shown). Inactivation of apoptotic pathways is often critical for the pathogenesis of tumor cells and for their resistance to chemotherapeutic drug treatment and/or irradiation [51-53]. It needs to be determined if the loss of CASP8 and/or maspin expression can be a prognostic marker for Breast Cancer and if it is associated with amplification of MYCN (v-myc myelocytomatosis viral related oncogene), as frequently observed in neuroblastomas.

Limitations of this study are that the most investigation was used cell lines only. To better understand the clinical relevance of CASP8 and/or maspin promoter methylation in breast tumors and if the decreased mRNA expression of CASP8 and/or maspin were correlated to the aberrant pattern of cytosine methylation of the gene promoter further studies with large sample size should be conducted.

## Conclusions

In conclusion, our results show that methylation of CpG sites at the promoter region in certain genes, such as CASP8 and maspin, could result in transcriptional downregulation or silencing of genes and protein in breast cancer cells. The anticancer drug, 5-Fu, is able to upregulate CASP8 gene expression, and the mechanism may involve demethylation. Screening promoter methylation patterns in breast cancer patients could be an important step to develop treatment protocols that target the methylated gene and improve DFS in breast cancer patients. Future studies also should examine the use of non-toxic demthylating agents in combination with chemotherapeutic drugs would offer an advantage for the treatment of breast cancer patients.

## Abbreviations

CASP8: Caspase 8; DNMT: DNA Methyltransferase; MSP: Methylated Specific Primer; 5-azadc: 5-aza-2'-deoxycytidine; TSA: Trichostatin A; HDAC: H; 5-Fu: 5-Fluorouracil; ATCC: American Type Culture Collection (Manassas, VA, USA); ER: estrogen receptor; IHC: Immunohistochemistry

## Competing interests

The authors declare that they have no competing interests.

## Authors' contributions

YW, MA and JVV were responsible for data collection, analysis, manuscript preparation, and editing. HPK and DS critical review and were involved in study design. All authors read and approved the final manuscript.

## Pre-publication history

The pre-publication history for this paper can be accessed here:

http://www.biomedcentral.com/1471-2407/10/32/prepub

## Supplementary Material

Additional file 1 **Figure S1. Promoter CpG sites of CASP8 and maspin and the primers sequences covered regions**. A figure to show the primers sequences used for MSP and bisulfate sequence covered regions in CASP8 promoter. Figure Legend for Figure S1. Click here for file
