# Supplementary material for: Caspase 8 and maspin are downregulated in breast cancer cells due to CpG site promoter methylation
Source: BMC Cancer. 2010 Feb 4;10:32. doi: 10.1186/1471-2407-10-32 (PMC2824712; doi:10.1186/1471-2407-10-32)
Supplement: Additional file 1 — Figure S1. Promoter CpG sites of CASP8 and maspin and the primers sequences covered regions. A figure to show the primers sequences used for MSP and bisulfate sequence covered regions in CASP8 promoter. Figure Legend for Figure S1. [file 1471-2407-10-32-S1.PPT]

## Slide 1
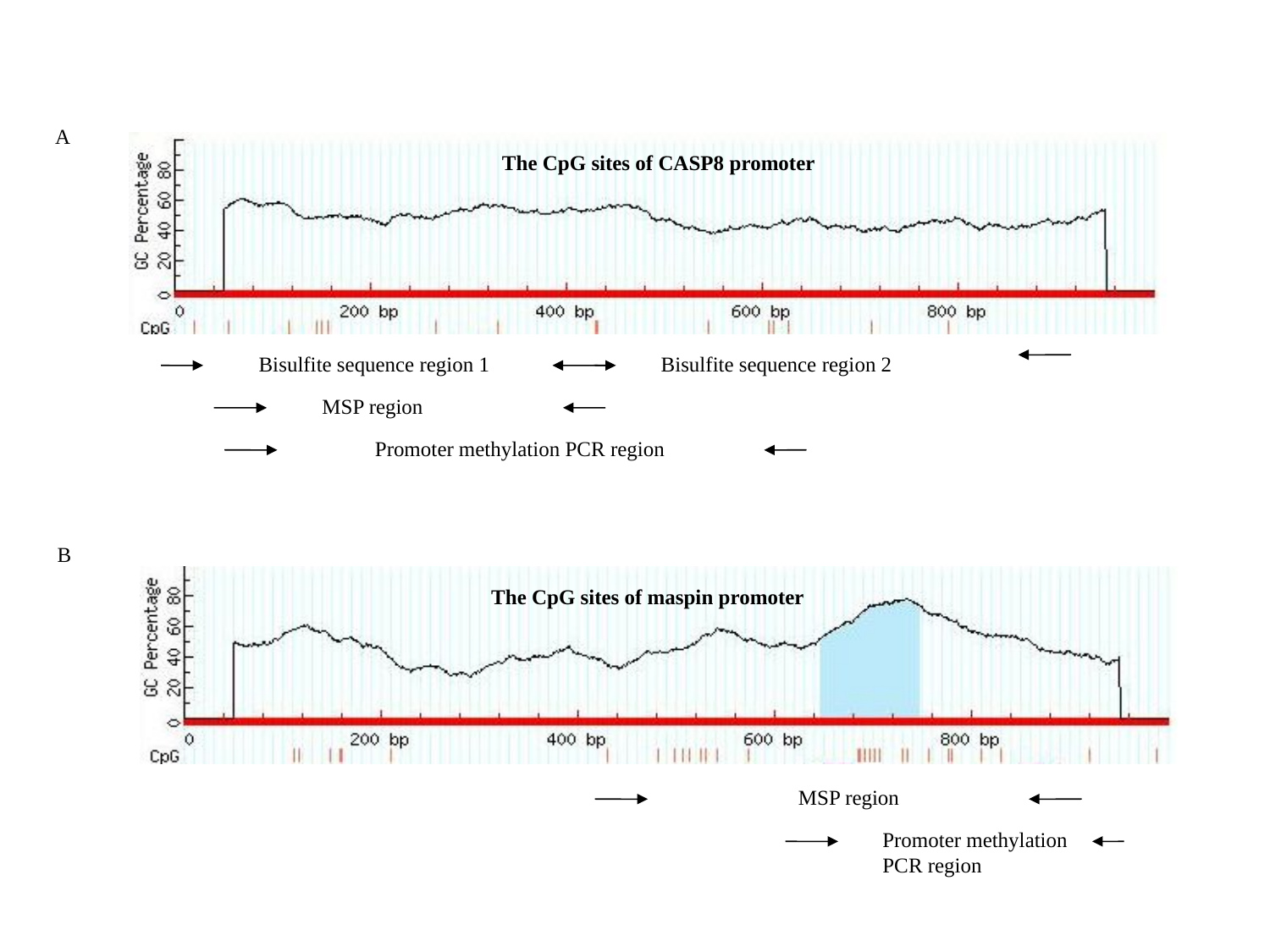

A
The CpG sites of CASP8 promoter
Bisulfite sequence region 1
Bisulfite sequence region 2
MSP region
Promoter methylation PCR region
B
The CpG sites of maspin promoter
MSP region
Promoter methylation PCR region

## Slide 2
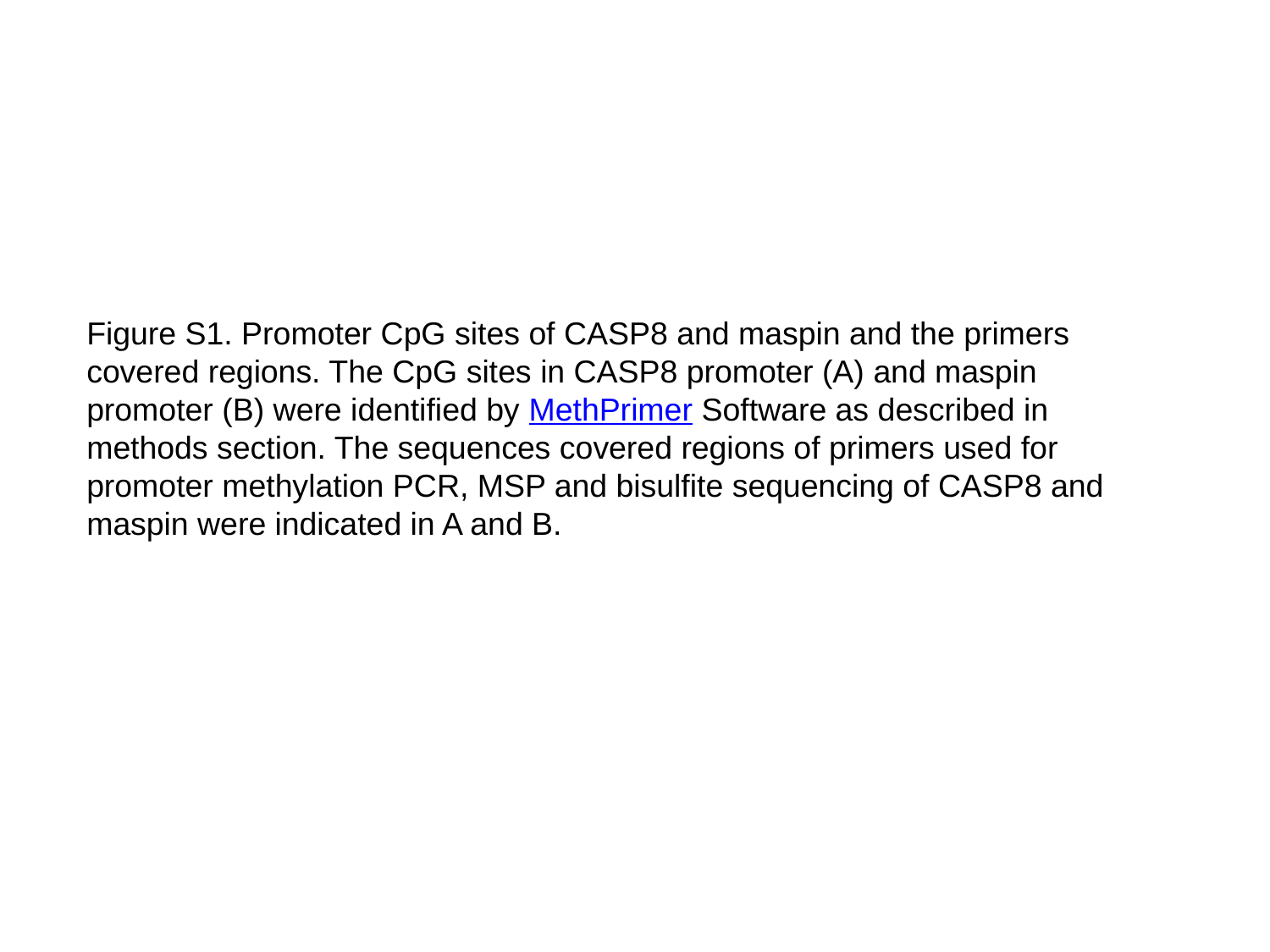

Figure S1. Promoter CpG sites of CASP8 and maspin and the primers covered regions. The CpG sites in CASP8 promoter (A) and maspin promoter (B) were identified by MethPrimer Software as described in methods section. The sequences covered regions of primers used for promoter methylation PCR, MSP and bisulfite sequencing of CASP8 and maspin were indicated in A and B.
